# SUMO Modification Regulates BLM and RAD51 Interaction at Damaged Replication Forks

**DOI:** 10.1371/journal.pbio.1000252

**Published:** 2009-12-01

**Authors:** Karen J. Ouyang, Leslie L. Woo, Jianmei Zhu, Dezheng Huo, Michael J. Matunis, Nathan A. Ellis

**Affiliations:** 1 Committee on Genetics, Genomics, and Systems Biology, University of Chicago, Chicago, Illinois, United States of America; 2 Department of Biochemistry and Molecular Biology, University of Chicago, Chicago, Illinois, United States of America; 3 Bloomberg School of Public Health, Department of Biochemistry and Molecular Biology, Johns Hopkins University, Baltimore, Maryland, United States of America; 4 Department of Health Studies, University of Chicago, Chicago, Illinois, United States of America; 5 Department of Medicine, University of Chicago, Chicago, Illinois, United States of America; Brandeis University, United States of America

## Abstract

The gene mutated in Bloom's syndrome, *BLM*, is important in the repair of damaged replication forks, and it has both pro- and anti-recombinogenic roles in homologous recombination (HR). At damaged forks, BLM interacts with RAD51 recombinase, the essential enzyme in HR that catalyzes homology-dependent strand invasion. We have previously shown that defects in BLM modification by the small ubiquitin-related modifier (SUMO) cause increased γ-H2AX foci. Because the increased γ-H2AX could result from defective repair of spontaneous DNA damage, we hypothesized that SUMO modification regulates BLM's function in HR repair at damaged forks. To test this hypothesis, we treated cells that stably expressed a normal BLM (BLM+) or a SUMO-mutant BLM (SM-BLM) with hydroxyurea (HU) and examined the effects of stalled replication forks on RAD51 and its DNA repair functions. HU treatment generated excess γ-H2AX in SM-BLM compared to BLM+ cells, consistent with a defect in replication-fork repair. SM-BLM cells accumulated increased numbers of DNA breaks and were hypersensitive to DNA damage. Importantly, HU treatment failed to induce sister-chromatid exchanges in SM-BLM cells compared to BLM+ cells, indicating a specific defect in HR repair and suggesting that RAD51 function could be compromised. Consistent with this hypothesis, RAD51 localization to HU-induced repair foci was impaired in SM-BLM cells. These data suggested that RAD51 might interact noncovalently with SUMO. We found that in vitro RAD51 interacts noncovalently with SUMO and that it interacts more efficiently with SUMO-modified BLM compared to unmodified BLM. These data suggest that SUMOylation controls the switch between BLM's pro- and anti-recombinogenic roles in HR. In the absence of BLM SUMOylation, BLM perturbs RAD51 localization at damaged replication forks and inhibits fork repair by HR. Conversely, BLM SUMOylation relieves its inhibitory effects on HR, and it promotes RAD51 function.

## Abstract 2

SUMO modification of BLM controls the switch between BLM's pro- and anti-recombinogenic roles in homologous recombination following DNA damage during replication.

## Author Summary

Replication is the process in which cellular DNA is duplicated. DNA damage incurred during replication is detrimental to the cell. Homologous recombination, in which DNA sequences are exchanged between two similar or identical strands of DNA, plays a pivotal role in correcting replication processes that have failed due to DNA breakage and is tightly regulated, because deficient or excess recombination results in genomic instability. Previous studies have implicated the DNA-processing enzyme BLM in the regulation of homologous recombination; BLM is defective in Bloom's syndrome, which is characterized by excess recombination and cancer susceptibility. Here, we show that modification of BLM by the small protein SUMO controls BLM's function in regulating homologous recombination at sites where DNA replication failed. We showed that cells expressing a SUMO-deficient mutant of BLM accumulated more DNA damage and displayed defects in repair by homologous recombination. An enzyme involved in homologous recombination, RAD51, displayed a defect in localization to sites where DNA replication failed. Our data support a model in which SUMO modification regulates BLM's function in homologous recombination by controlling the localization of RAD51 to failed replication sites.

## Introduction

Homologous recombination (HR) is a high-fidelity DNA repair mechanism that functions to rejoin double-strand breaks (DSBs) and restart broken replication forks. A major outcome of the repair of replication fork damage by HR is the generation of sister-chromatid exchanges (SCEs), which result from resolution of Holliday junctions during HR repair [1],[2]. Predictably, a large number of agents that cause DNA damage increase the frequencies of SCEs [3]–[5]. Bloom's syndrome (BS) is the only clinical entity in which increased levels of SCE are a prominent cellular feature [6]. It is an autosomal recessive disorder, which is characterized by proportional dwarfism, photosensitivity, immunodeficiency, hypogonadism, and predisposition to a wide range of different types of cancer [7]. BS is caused by biallelic null mutations of the *BLM* gene [8]. The *BLM* gene encodes a DNA helicase of the RecQ family, which is an evolutionarily conserved group of enzymes that operates at the interface of DNA replication, HR, and DNA repair [9].

The RecQ helicases are DNA-dependent ATPases that can translocate on single-stranded DNA (ssDNA) with 3′ to 5′ directionality [10]. In vitro, they preferentially unwind DNA substrates that resemble recombination intermediates, including G4 tetrahelical DNA, Holliday junctions, double Holliday junctions, and D-loops. A complex consisting of BLM, topoisomerase IIIα, BLAP75, and BLAP18 (BLAPs are BLM-associated proteins) can “dissolve” a substrate representing a double Holliday junction—a late intermediate in HR-mediated repair of DSBs—in such a way that crossing over would not occur between DNA strands [11]–[13]. This activity could provide an explanation for the increased SCEs in BS cells; however, recent genetic and biochemical studies have shown that BLM also has activities in upstream parts of the HR pathway. Because BLM is recruited to damaged replication forks early in the repair process [14]–[16], it could suppress the formation of aberrant recombination intermediates at the replication fork. Such a mechanism has been proposed for Sgs1, the yeast homolog of BLM [17],[18]. BLM interacts directly with the RAD51 recombinase, which is the enzyme that catalyzes homology-dependent strand invasion [19],[20], and in vitro it can displace RAD51 from ssDNA and unwind the invading DNA strand of a D-loop formed by RAD51 [21],[22], suggesting that BLM regulates the formation of D loops. Finally, BLM and Sgs1 each collaborate with exonucleases that process DSBs to generate ssDNA with a 3′ tail, which is the substrate for RAD51 [23]–[26]. Collectively, these data show that BLM has both pro- and anti-recombinogenic functions in HR. A key question that emerges from these studies is how are these different functions of BLM in HR regulated?

Modification by the small ubiquitin-related modifier (SUMO) has emerged as an important regulator of HR [27]. In response to replication fork damage in the budding yeast *Saccharomyces cerevisiae*, the polymerase processivity factor PCNA (proliferating cell nuclear antigen) is SUMOylated, and PCNA SUMOylation recruits the DNA helicase Srs2 to the fork, which functions to prevent aberrant recombination events between sister chromatids [28]–[31]. Mutants of the SUMO-specific E3 ligase gene *MMS21* accumulate RAD51-dependent cruciform structures at damaged replication forks [32], which are aberrant structures that also accumulate in *sgs1* deletion mutants [33]. These studies indicate that SUMO modification can play important roles in response to damaged forks; however, the role of SUMO in regulation of HR is not fully understood, and these mechanisms have not been studied in mammalian cells.

We have previously shown that BLM is SUMOylated and that failure to SUMOylate BLM results in changes in BLM's nuclear distribution [34]. Expression in cells of SUMO-mutant BLM, containing lysine to arginine mutations at residues 317 and 331 that prevent SUMOylation, induces excess γ-H2AX foci—a marker for DNA damage and repair—in the absence of exogenously induced DNA damage [34]. Despite the presence of excess γ-H2AX foci and micronuclei in cells that expressed SUMO-mutant BLM, there was insufficient evidence to conclude that SUMO-mutant BLM generated excess DNA damage. Because SUMOylation is known to regulate the localization of proteins in the nucleus [35], we hypothesized that the accumulation of BLM in γ-H2AX foci could result from a kinetic defect in recruitment of BLM back to the promyelocytic leukemia (PML) nuclear bodies (PML-NBs).

In the present study, we aimed to determine how SUMOylation regulates BLM's function in maintaining genomic integrity. We hypothesized that cells that express SUMO-mutant BLM have a DNA repair defect. Characterization of cells that expressed SUMO-mutant BLM revealed that SUMOylation of BLM regulates its association with RAD51 and its function in HR-mediated repair of damaged replication forks. Our data support a model in which SUMOylation of BLM acts as a switch to regulate its effects on recombination.

## Results

### Excess γ-H2AX at Damaged Replication Forks in SUMO-Mutant BLM Cells

**Figure 1:**
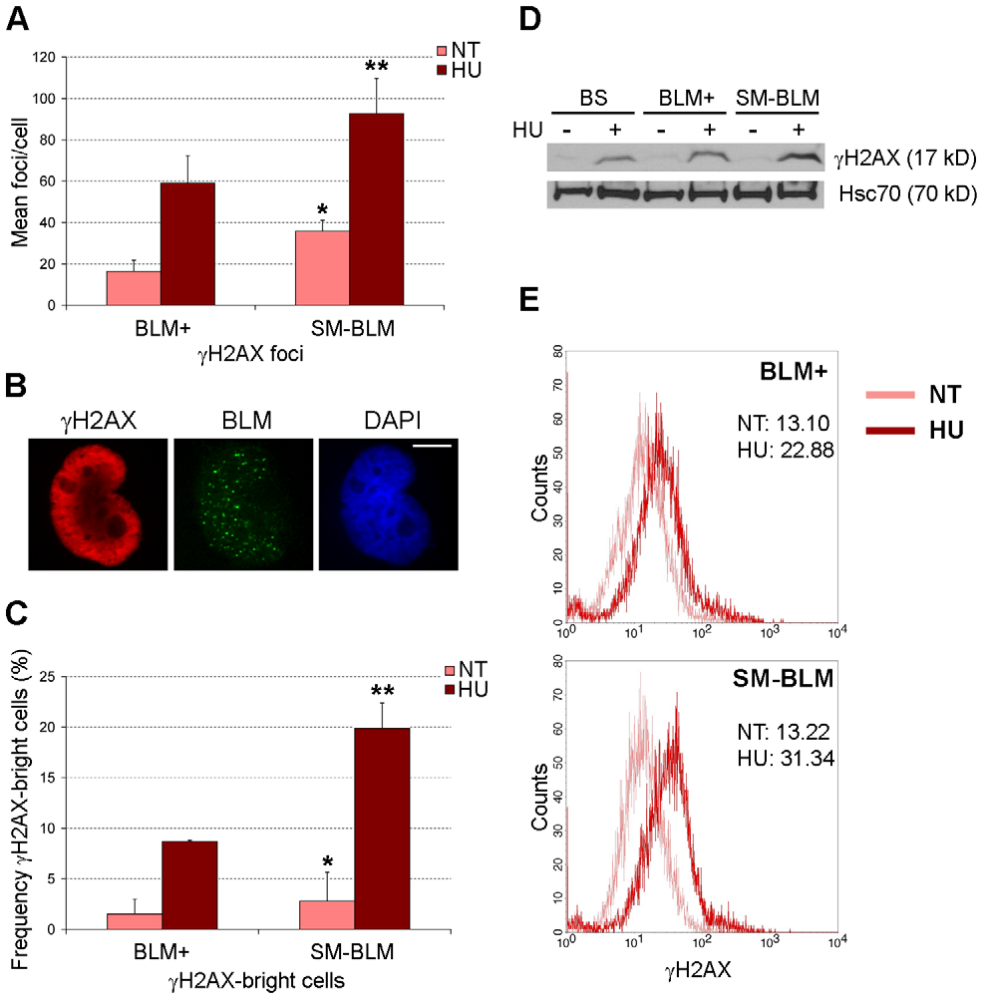
HU treatment induces more γ-H2AX in SM-BLM compared to BLM+ cells. (A) Graphical representation of the average numbers of γ-H2AX foci in HU-treated and untreated BLM+ and SM-BLM cells. BLM+ and SM-BLM cells were untreated (NT) or treated with 0.5 mM HU for 24 h (HU) and then processed for immunofluorescence. Data presented are the average of three experiments in three BLM+ and three SM-BLM clones. Approximately 35 cells were collected in each experiment. Error bars represent standard deviations of the combined data. *p*-Values were calculated using mixed effects linear models as described in Materials and Methods. *p = 0.02, NT SM-BLM versus NT BLM+; **p = 0.02, HU-treated SM-BLM versus HU-treated BLM+. (B) Excess γ-H2AX-bright cells accumulate in HU-treated SM-BLM cells. Immunofluorescence images of a representative γ-H2AX-bright cell stained with antibodies to γ-H2AX (left panel) and with DAPI (right panel), showing GFP-BLM fluorescence (center panel). SM-BLM localizes to many discrete foci in γ-H2AX-bright cells. Bar indicates 10 µm. (C) Graphical representation of percentages of γ-H2AX-bright cells in BLM+ and SM-BLM cells after no treatment (NT) or treatment (HU) with 0.5 mM HU for 24 h. Error bars represent standard deviations of the combined data. *p = 0.06, NT SM-BLM versus NT BLM+; **p<10^−3^, HU-treated SM-BLM versus HU-treated BLM+. (D) Western blot analysis of γ-H2AX levels in HU-treated and untreated cells. Hsc70 is a control for protein loading. (E) Flow cytometry analysis of γ-H2AX-stained BLM+ and SM-BLM cells. Inset numerical values denote median fluorescence intensities in arbitrary units.

Because BLM functions at damaged replication forks and γ-H2AX is a marker for DNA damage, we hypothesized that SUMO-mutant BLM is defective in repair of damaged replication forks. To gain insight into this question, we introduced GFP-BLM expression constructs into the BS cell line GM08505, isolated clones that stably expressed either normal BLM (BLM+ cells) or SUMO-mutant BLM (SM-BLM cells), and studied these clones for responses to replication fork damage. We treated SM-BLM and BLM+ cells with 0.5 mM hydroxyurea (HU) for 24 h, which stalls replication forks, and quantified the production of γ-H2AX by immunofluorescence, Western blot, and flow cytometry analyses (Figure 1). The 24-h HU treatment blocks approximately 80% of the cells in S phase, simultaneously providing a primary synchronization of the cells and stressing replication forks through nucleotide deprivation.

As we reported previously, untreated SM-BLM cells exhibited higher levels of γ-H2AX foci compared to untreated BLM+ cells (35.9 vs. 16.4, respectively; Figure 1A). Treatment of SM-BLM and BLM+ cells with HU resulted in a larger increase in γ-H2AX foci per cell in SM-BLM compared to BLM+ cells (a gain of 56.8 vs. 42.7 foci). Particularly notable were the presence of SM-BLM cells that stained brightly with γ-H2AX (Figure 1B). HU induced twice the numbers of γ-H2AX-bright nuclei in SM-BLM cells than in BLM+ cells (Figure 1C). Consistent with the immunofluorescence analysis, by Western blot and flow cytometry analyses, the levels of γ-H2AX in HU-treated SM-BLM cells were higher than in HU-treated BLM+ cells (Figure 1D and 1E).

Because the results could have been influenced by cell-cycle effects, we analyzed nuclear DNA content and measured BrdU incorporation at different times after release from the HU block by flow cytometry. After treatment with HU, in both SM-BLM and BLM+ clones, the majority of cells were blocked in early S phase, and they progressed to mid-S phase by 6 h after release from the HU block (Figure S1). These data indicated that differences in position in the cell cycle or irreversibility of the S phase block did not explain the differences in the accumulation of γ-H2AX after HU treatment of SM-BLM and BLM+ cells.

In summary, these experiments showed that SM-BLM cells exhibited excess phosphorylated H2AX in both untreated and HU-treated conditions. The presence of increased levels of spontaneous and HU-induced γ-H2AX strongly suggests the presence of excess DNA damage.

### Replication Fork Damage Induces Increased DSBs in SUMO-Mutant BLM Cells

**Figure 2:**
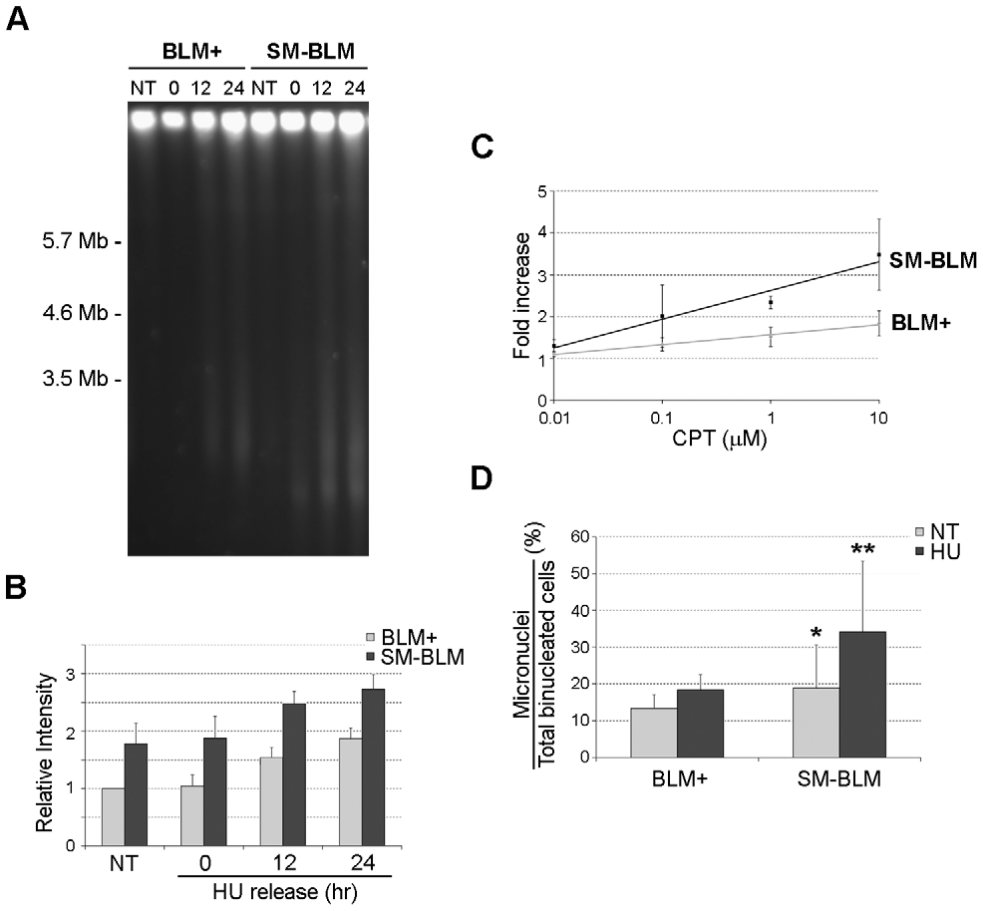
SUMO-mutant BLM cells accumulate excess DSBs at damaged replication forks. (A) Pulsed-field gel showing the analysis of untreated BLM+ and SM-BLM cells (NT) and cells treated with HU for 24 h (0 time) followed by release into normal medium for 12 and 24 h. DSBs were visualized by DNA fragments that migrate into the gel, whereas intact DNA was retained in the well. (B) Quantification of DSBs after treatment with HU and release into normal medium. The bars represent the numbers of DSBs relative to untreated BLM+ cells in two independent experiments with two clones of each type, and the error bars represent the standard deviations of the combined data. (C) Quantification of accumulation of DSBs after treatment with different concentrations of CPT for 3 h. Data points represent the numbers of DSBs relative to untreated BLM+ or SM-BLM cells from a minimum of two experiments with two clones of each type. Error bars represent standard deviations of the combined data. (D) Quantification of HU-induced micronuclei. Cells were untreated or treated with 0.5 mM HU for 24 h, followed by incubation with cytochalasin-B for 28 h in the absence of HU. The numbers of micronuclei were counted in binucleated cells. A minimum of 500 binucleated cells were assessed under each condition. Data presented are the average of three experiments in three BLM+ and three SM-BLM clones. The bars represent mean numbers of micronuclei, and error bars represent standard deviations of the combined data. *p = 0.6, NT SM-BLM versus NT BLM+; **p<10^−3^, HU-treated SM-BLM versus HU-treated BLM+.

In order to obtain direct evidence for the presence of DNA damage, we analyzed HU-treated and untreated cells for DSBs by pulsed-field gel electrophoresis (PFGE) (Figure 2). In the absence of treatments, SM-BLM cells exhibited over 1.5 times more DSBs compared to BLM+ cells (Figure 2B). This result confirmed the presence of increased numbers of DSBs consistent with the higher numbers of γ-H2AX foci. After a 24-h treatment with HU, SM-BLM cells again exhibited 1.5 times more DSBs compared to BLM+ cells. Because 80% of the cells are blocked with stalled forks, the DSBs detected in HU treatment conditions likely originate from fork breakage. After release from the HU block, DSBs accumulated over time, with the total number of DSBs observed in SM-BLM cells being greater at each time point than the total number in BLM+ cells (Figure 2B). A 24-h HU treatment induced more DSBs in BS cells compared to no treatment, and BS cells also accumulated more DSBs after release from the HU block compared to either SM-BLM or BLM+ cells (Figure S2). Consistent with the PFGE analysis, the total number of HU-induced micronuclei was greater in SM-BLM compared to BLM+ cells (Figure 2D).

We also analyzed the numbers of DSBs in cells treated with camptothecin (CPT), which generates replication-associated DSBs [36],[37]. Treatment with different concentrations of CPT for 3 h generated two times more DSBs in SM-BLM compared to BLM+ cells, showing that breaks accumulate at an accelerated rate in SM-BLM cells (Figure 2C). Altogether, these data were consistent with the hypothesis that SM-BLM cells have a defect in the repair of replication-associated DSBs.

### Replication Fork Damage Induces Increased Cell Death in SUMO-Mutant BLM Cells

**Figure 3:**
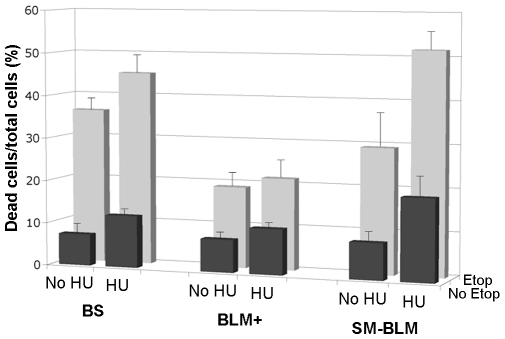
SM-BLM cells are hypersensitive to DNA damage encountered during S phase. Cells were untreated (NT) or treated with 0.5 mM HU for 24 h and then untreated or treated with 50 µM etoposide for 24 h. Cell viability was measured using the Guava ViaCount reagent, and cell death was calculated as the percentage of dead cells divided by the total number of cells. The bars represent the mean percentage of dead cells in each condition in three BLM+ clones and three SM-BLM clones from a minimum of three independent experiments performed in triplicate. Error bars represent standard deviations of the combined data. See text for *p*-values of relevant comparisons.

Because SM-BLM cells exhibited higher levels of DSBs induced by replication damage, we expected SM-BLM cells to be hypersensitive to DNA damage encountered during S phase. To test this hypothesis, we compared the levels of cell death in cells exposed to replication damage, using a standard flow cytometry assay (Figure 3). In the absence of HU or etoposide treatment, BLM+ and SM-BLM cells exhibited similar levels of cell death. A 24-h treatment with HU induced a 3% increase in cell death in BLM+ cells as compared to a 10.6% increase in SM-BLM cells (*p* = 0.01), demonstrating that SM-BLM cells have increased sensitivity to HU treatment alone. Similarly, a 24-h treatment with etoposide induced an 11.3% increase in cell death in BLM+ cells compared to a 20.7% increase in SM-BLM cells (*p* = 0.003), demonstrating that SM-BLM cells are also hypersensitive to etoposide treatment compared to BLM+ cells. After HU pretreatment, etoposide induced a 13.5% increase in cell death in BLM+ cells, which was similar to the level of cell death observed without HU pretreatment (11.3%) (*p* = 0.84), whereas after HU pretreatment, etoposide induced a 43.1% increase in cell death in SM-BLM cells, which was 2-fold greater compared to the level observed without HU pretreatment (20.7%) (*p*<0.001). As expected, BS cells that lack BLM protein are also hypersensitive to DNA damage encountered during S phase. In corroboration of these results, in colony survival assays, we also observed increased sensitivity of SM-BLM cells to CPT compared to BLM+ cells (Figure S3). These data indicated that SM-BLM cells are more sensitive than BLM+ cells to DNA damage generated during S phase, again consistent with a defect in the repair of replication-associated DNA damage.

### Replication Fork Damage Fails to Induce HR in SUMO-Mutant BLM Cells

**Figure 4:**
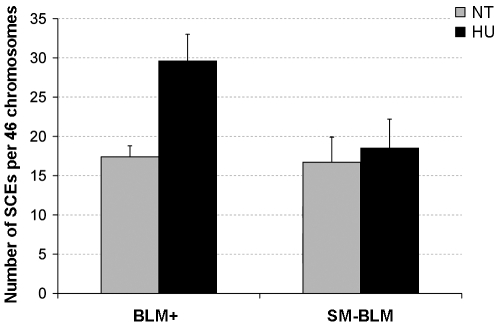
Cells that express SUMO-mutant BLM do not exhibit increased HU-induced SCEs. BrdU was incorporated for one cell-division cycle prior to HU treatment. Cells were treated with 0.5 mM HU for 24 h and then released into BrdU-containing medium for another 20 h. Metaphases were collected in colcemid. The bars represent the mean numbers of SCEs in two BLM+ and two SM-BLM clones from a minimum of three independent experiments. Error bars represent standard deviations of the combined data. See text for *p*-values of relevant comparisons.

Replication-associated DSBs are repaired by HR, which generates increased numbers of SCEs [1]. Because SM-BLM cells exhibited excess HU-induced DSBs, we hypothesized that HR is impaired in SM-BLM cells. Therefore, we tested whether replication stalling induced fewer SCEs in SM-BLM compared to BLM+ cells (Figure 4). Untreated BLM+ and SM-BLM cells showed similar numbers of SCEs (17.4 vs. 16.7 SCEs/46 chromosomes, respectively). However, whereas HU treatment induced a 2-fold increase in SCEs in BLM+ cells (from 17.4 to 29.6 SCEs/46 chromosomes; *p*<0.001), HU treatment had almost no effect on the levels of SCEs in SM-BLM cells (from 16.7 to 18.5 SCEs/46 chromosomes; *p* = 0.32). The numbers of HU-induced SCEs in BLM+ compared to SM-BLM cells was significantly different (*p*<0.001). In contrast to SM-BLM cells, HU induced a large increase in SCEs in BS cells (Figure S4A). These data suggest that HR repair is not engaged normally at damaged replication forks, leading to the excess DSBs that are observed in HU-treated SM-BLM cells.

It is worth noting that in our earlier report on BLM SUMOylation [34], we found that the mean number of SCEs in untreated SM-BLM cells was greater than the mean number in BLM+ cells. This difference was caused by the presence in the SM-BLM cultures of 5% of cells with SCE levels equal to levels typically observed in BS cells, whereas we had detected no cells of this type in the BLM+ cultures. In the present study, we did not detect cells with high SCEs in the SM-BLM cultures; consequently, we suggest that these high-SCE cells were produced by extinction of SM-BLM expression in a small fraction of SM-BLM cells.

### Localization of RAD51 to Damaged Forks Is Defective in SUMO-Mutant BLM Cells

**Figure 5:**
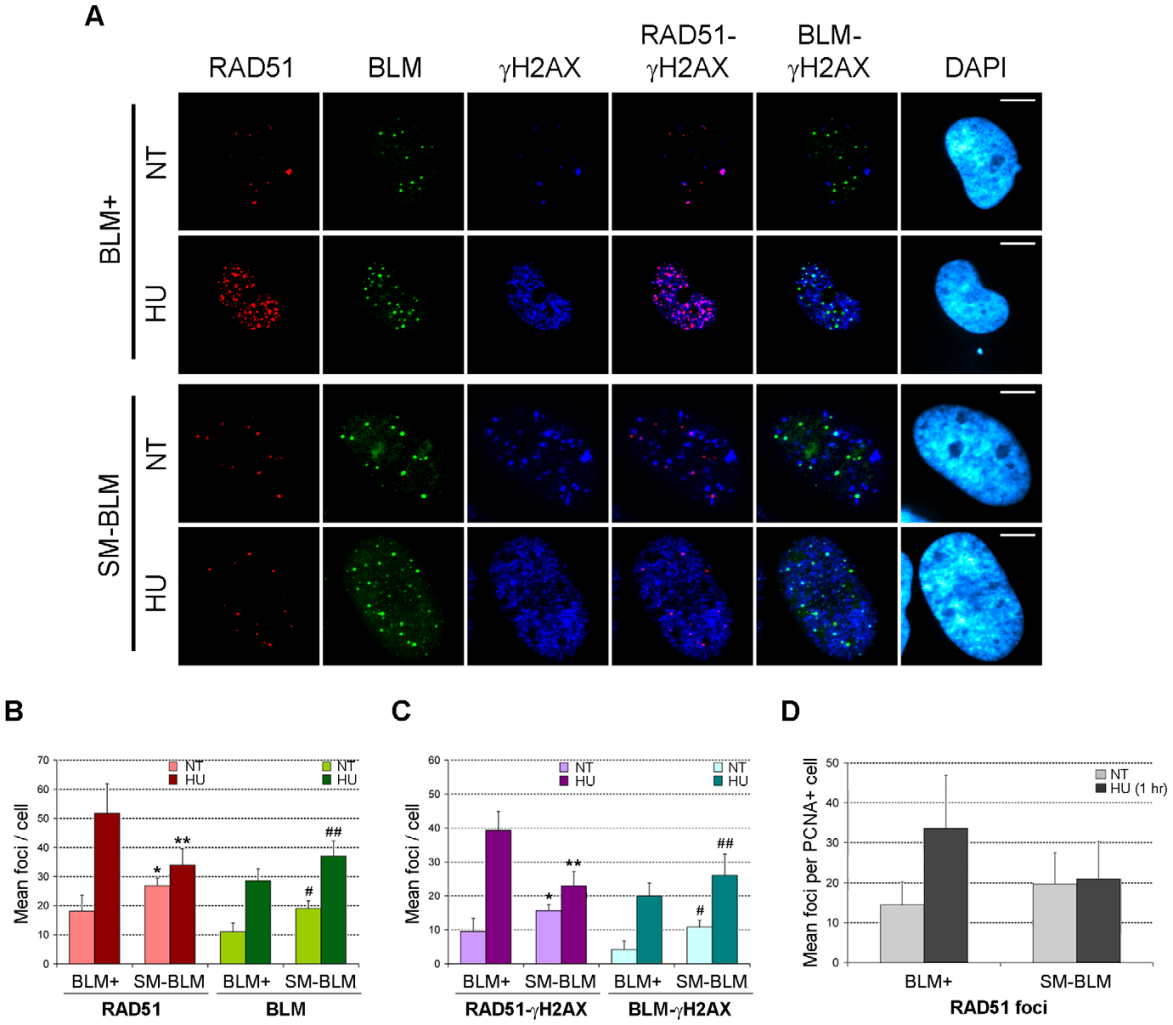
RAD51 has a localization defect in HU-treated SM-BLM cells. (A) Immunofluorescence images of BLM+ and SM-BLM cells treated with 0.5 mM HU for 24 h or not treated (NT) and stained for γ-H2AX and RAD51. Bars indicate 10 µm. (B) Graphical representation of mean numbers of RAD51 and BLM foci in HU-treated and untreated BLM+ and SM-BLM cells. *p = 0.09, NT SM-BLM versus NT BLM+; **p = 0.02, HU-treated SM-BLM versus HU-treated BLM+. #*p* = 0.01, NT SM-BLM versus NT BLM+; ##*p* = 0.01, HU-treated SM-BLM versus HU-treated BLM+. (C) Graphical representation of mean numbers of colocalized RAD51-γ-H2AX and colocalized BLM-γ-H2AX foci in HU-treated and untreated BLM+ and SM-BLM cells. *p<10^−3^, NT SM-BLM versus NT BLM+; **p<10^−3^, HU-treated SM-BLM versus HU-treated BLM+; #*p*<10^−3^, NT SM-BLM versus NT BLM+; ##*p* = 0.02, HU-treated SM-BLM versus HU-treated BLM+. Bars in (B) and (C) represent the average foci in three independent experiments of three BLM+ and three SM-BLM clones. Error bars represent the standard deviations of the combined data. (D) Graphical representation of means numbers of RAD51 foci in untreated cells or cells treated with 10 mM HU for 1 h. RAD51 foci were counted in cells that stained positively with PCNA antibodies. Bars represent the average foci in two independent experiments of two BLM+ and two SM-BLM clones. Error bars represent the standard deviations of the combined data.

RAD51 is a key enzyme in HR repair, and normally it interacts with BLM at damaged replication forks [19],[20]. Because SM-BLM cells exhibited a defect in HR-mediated repair after replication stalling, we examined whether RAD51 and BLM colocalize normally at γ-H2AX–marked damage in SM-BLM cells (Figure 5). In untreated cells, SM-BLM cells contained more RAD51 foci than BLM+ cells (26.9 vs. 18.1 foci/cell). Similarly, as previously noted [34], untreated SM-BLM cells contained more BLM foci (19.0 vs. 11.0 foci/cell) (Figure 5B). However, after treatment with 0.5 mM HU for 24 h, whereas BLM+ cells exhibited a large increase in RAD51 foci from 18.1 to 51.7 foci/cell, SM-BLM cells exhibited only a modest increase from 26.9 to 34 foci/cell (Figure 5B). Consistent with these observations, whereas HU treatment induced substantial increases in RAD51-γ-H2AX and RAD51-BLM colocalization in BLM+ cells, HU treatment induced only a modest increase in these colocalizations in SM-BLM cells (Figure 5C and Figure S5). In HU-treatment conditions, whereas 66% of the γ-H2AX foci contained RAD51 in BLM+ cells (vs. 58% in untreated cells), only 25% of γ-H2AX foci contained RAD51 in SM-BLM cells (vs. 43% in untreated cells—a decrease). As a positive control, we found that RAD51 showed much higher levels of colocalization with γ-H2AX in HU-treated BS cells compared to BLM+ cells (Figure S4B), as previously reported [19].

To distinguish whether the RAD51 localization defect was an early or late effect of replication stalling, we treated BLM+ and SM-BLM cells with 10 mM HU for 1 h and stained cells with antibodies to RAD51 and PCNA (to identify cells in S phase). Whereas BLM+ cells exhibited a 2.3-fold increase in RAD51 foci from 14.4 to 33.6 foci/cell in PCNA-positive cells, SM-BLM cells exhibited no increase in total RAD51 foci from 19.7 to 21.0 foci/cell (Figure 5D). Synchronization experiments with mimosine followed by treatment with 10 mM HU for 1 h corroborated these data (Figure S6). It is worth noting that both BLM+ and SM-BLM proteins localized efficiently with PCNA foci in HU-treated cells, indicating that SUMOylation is not required for normal trafficking of BLM to stalled forks (Figure S7).

Altogether, these data demonstrated that there is a dramatic defect in RAD51's recruitment to and/or retention in repair foci induced by replication stalling. The RAD51 localization defect could explain both the impairment of HR after replication stalling and the excess DSBs observed in SM-BLM cells.

### RAD51 Binds SUMOylated BLM

**Figure 6:**
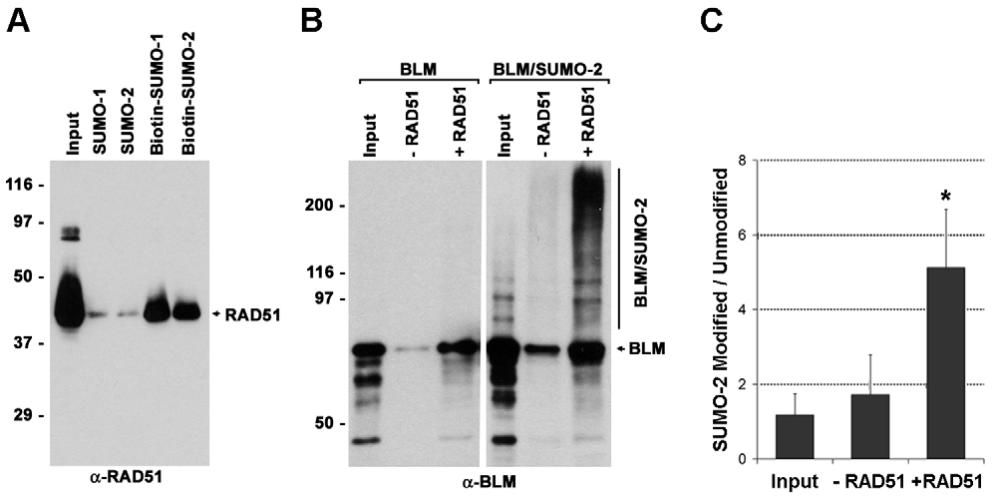
RAD51 contains a SUMO binding site. (A) Biotinylated SUMO-1 and SUMO-2 were bound to streptavidin beads and incubated with purified recombinant, human RAD51; the complexes were pulled down and analyzed by Western blot with anti-RAD51. As a control, unbiotinylated SUMO-1 and SUMO-2 were incubated with beads and incubated with RAD51. (B) A purified, recombinant N-terminal fragment of BLM, consisting of amino acids 1–431, was modified with SUMO-2 in vitro. Equal amounts of SUMOylated or unSUMOylated BLM were incubated with RAD51 bound to streptavidin beads, and complexes were pulled down and analyzed by Western blot with anti-BLM. As a control, SUMOylated or unSUMOylated BLM was incubated with unconjugated streptavidin beads. (C) Ratios of SUMO-2–modified BLM to unmodified BLM in input fraction and fractions bound to control beads and beads containing RAD51. Experiment was performed three times. *p = 0.03, RAD51-coated beads versus control beads.

To investigate the mechanism that might explain the RAD51 localization defect, we considered the possibility that RAD51 interacts with SUMO noncovalently, which would facilitate interaction between RAD51 and SUMOylated BLM. To test this hypothesis, we assayed for possible noncovalent interactions between RAD51 and SUMO and also for possible effects of covalent SUMOylation of BLM on its interactions with RAD51 (Figure 6). In an in vitro binding assay, more RAD51 bound to SUMO-coated beads than to controls beads (Figure 6A), showing that RAD51 binds equally well to both SUMO-1 and SUMO-2. To test whether SUMO modification of BLM affects its interaction with RAD51, we incubated RAD51-coated beads with either unmodified BLM or with a mixture of SUMO-2–modified and unmodified BLM and analyzed bound proteins by Western blot with anti-BLM antibodies. Consistent with previous findings [20], unmodified BLM bound specifically to RAD51-coated beads, confirming that BLM and RAD51 interact directly (Figure 6B, left panel). SUMO-2–modified BLM also bound specifically to RAD51-coated beads (Figure 6B, right panel). To evaluate the effect of SUMO-2 modification on BLM's interaction with RAD51, the results of the binding assays were analyzed quantitatively. This analysis revealed that, whereas the ratio of SUMO-2–modified to unmodified BLM in input fractions and fractions bound to control beads was approximately 1.1∶1 and 1.7∶1, respectively, the ratio present in fractions retained on RAD51-coated beads was approximately 5.1∶1 (Figure 6C). These binding ratios reveal that SUMO-2 modification of BLM has a strong, positive effect on its binding to RAD51.

Altogether, these data demonstrated that RAD51 contains a SUMO binding site(s) and that SUMOylation of BLM can affect its interactions with RAD51. The data support the hypothesis that SUMOylation of BLM facilitates repair of damaged replication forks by HR by modulating RAD51's recruitment and/or retention at repair sites.

## Discussion

The data presented here demonstrate the importance of BLM SUMOylation in the repair of damaged replication forks. Failure to SUMOylate BLM resulted in excess damage-induced repair foci, DSBs, and hypersensitivity to DNA damage. Importantly, in SM-BLM cells, replication stalling by HU did not stimulate HR as measured by SCEs, suggesting a defect in RAD51 function. Consistent with these data, RAD51 failed to accumulate at stalled forks. SUMO-mutant BLM exerts a dominant effect, because excess γ-H2AX foci were induced by expression of SUMO-mutant BLM in HeLa cells, which express endogenous normal BLM [34]. Moreover, we showed here that several SM-BLM phenotypes differed from BS phenotypes, such as the presence of excess H2AX phosphorylation in untreated cells, the RAD51 localization defect, and the lack of HU-induced SCEs. We found that RAD51 is a SUMO-binding protein, implicating BLM SUMOylation in recruitment of RAD51 to repair sites through a mechanism involving noncovalent SUMO interactions. Our findings demonstrate that BLM SUMOylation regulates the recruitment and/or retention of RAD51 to damaged replication forks, and it is important in HR-mediated repair.

The steady-state levels of DSBs in cells are a function of the rate at which DNA damage accumulates and the rate at which it is repaired. We observed that SM-BLM cells exhibited greater numbers of DSBs than BLM+ cells under a variety of conditions (Figure 2). For example, SM-BLM cells exhibited more HU-induced DSBs, which arise due to breakage of stalled forks, and more CPT-induced DSBs, which arise due to replication runoff at sites where topoisomerase-cleavage complexes are bound to the DNA [38]. Formally, the presence of increased levels of DSBs in SM-BLM cells could result from an increase in the rate at which DNA damage accumulates (due to excess numbers of replication forks, increased numbers of topoisomerase cleavage complexes, or a failure to process aberrant replication intermediates) or from a decrease in the rate of DNA repair (due to a failure to recruit RAD51). We noted that the rate of DSB accumulation after release from the HU block was the same in both SM-BLM and BLM+ cells, indicating that the rate of breakage exceeds the rate of repair under these conditions in both types of cells. Consistent with these observations, the numbers of γ-H2AX foci in both BLM+ and SM-BLM cells increase 6 h after release from the HU block (unpublished data).

Recent work has shown that BLM is present on a class of ultrafine anaphase bridges [39], and it acts to separate interlinked DNA strands especially at loci with intrinsic replication difficulties [40]. BS cells consequently have a defect in separation of sister chromatids, resulting in more anaphase bridges; some fraction of the increased DSBs that arise in BS cells no doubt traces to breakage at sites of underreplicated DNA. BS cells exhibit an inadequate response to replication stress [16],[41]–[43], in which additional forks are initiated apparently to compensate for forks that have collapsed [44],[45]. One possible explanation for the excessive numbers of γ-H2AX foci and DSBs in treated and untreated SM-BLM cells is that these cells have increased replication difficulties, as BS cells do, and they compensate by activating additional replication forks, which are concomitantly more likely to break after replication damage. According to this view, BLM SUMOylation helps prevent the collapse of replication forks in regions with hard-to-replicate DNA, perhaps by stimulation of BLM's activity in subverting aberrant recombination intermediates at stalled replication forks.

Alternatively, BLM SUMOylation could promote HR-mediated repair of broken forks through the recruitment and/or retention of RAD51 at damaged forks. RAD51 is the DNA recombinase essential for HR-mediated DNA repair, and previous studies have demonstrated that RAD51 and BLM interact specifically in DNA-damaged cells [19],[20]. On the basis of immunolocalization studies, we found that the recruitment and/or retention of RAD51 at sites of stalled DNA replication forks is impaired in SM-BLM cells. Whereas BLM+ cells exhibited a 4-fold increase in the number of colocalized γ-H2AX-RAD51 foci upon HU treatment, SM-BLM cells exhibited a <1.5-fold increase in these foci. On the basis of this finding, and the finding that HR-mediated DNA repair is defective in SM-BLM cells, we propose that BLM SUMOylation mediates the recruitment and/or retention of RAD51 to sites of DNA damage and thereby facilitates HR-mediated repair processes.

**Figure 7:**
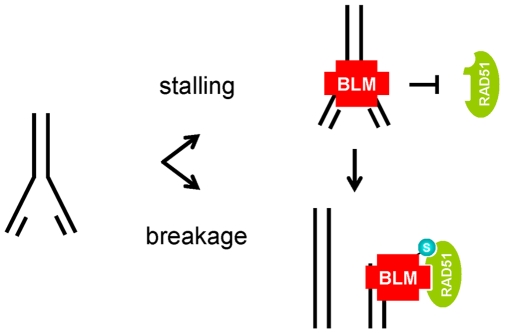
BLM SUMOylation regulates BLM and RAD51 function in HR-mediated repair of damaged forks. The model depicts a replication fork that has either stalled or broken. At stalled forks, unSUMOylated BLM inhibits access of RAD51 to the stalled fork. If the fork progresses to a DSB, BLM SUMOylation promotes the recruitment and/or retention of RAD51 to the broken end through noncovalent interactions between SUMOylated BLM and the SUMO binding site on RAD51. S, SUMO.

Previous cell and biochemical studies have led to the view that BLM has both pro- and anti-recombinogenic functions. Most notably, BLM is important in stabilizing damaged replication forks [14]–[16] and repressing aberrant recombination events, as evidenced by the dramatic increase in levels of SCEs and loss of heterozygosity in *BLM* null cells [6],[46]. In contrast, BLM is also predicted to promote HR by facilitating exonucleolytic resection of DSBs [23]–[26], by stimulating synthesis-dependent strand annealing [21],[47], and by promoting noncrossover resolution of Holliday junctions [12]. Both pro- and anti-recombinogenic functions have likewise been proposed for *Escherichia coli* RecQ helicase [48]. Our finding that cells expressing SUMO-mutant BLM have a defect in the recruitment and/or retention of RAD51 to sites of DNA damage, and that they are defective in HR-mediated repair, supports a model in which SUMOylation of BLM acts as a switch to regulate its effects on recombination (Figure 7). In the absence of SUMOylation, we propose that BLM binds to stalled replication forks and suppresses aberrant HR by inhibiting excessive accumulation of RAD51 at repair sites. In the event that a stalled replication fork progresses to a DSB, stimulation of HR-mediated repair would be triggered by BLM SUMOylation and, consequently, more efficient recruitment and/or retention of RAD51 at the repair site.

SUMOylation of BLM could regulate the recruitment and/or the retention of RAD51 at sites of DNA damage through several different mechanisms. SUMOylation could dissociate BLM from broken DNA ends, where it might otherwise inhibit RAD51 binding and function by displacing RAD51 from ssDNA or by unwinding D-loops [21],[22]. SUMOylation could limit the binding of BLM to sites of DNA damage by altering its affinity to ssDNA or possibly by triggering its ubiquitin-dependent degradation. SUMO-mediated degradation has been described for PML and other proteins [49]–[51]. In the model that we currently favor, BLM SUMOylation could function to promote RAD51 localization at repair sites by stabilizing its interactions with BLM through a mechanism involving noncovalent SUMO binding. Consistent with this model, we found that RAD51 is a SUMO-binding protein and that it interacts more efficiently with SUMOylated BLM compared to unmodified BLM. RAD51 is recruited to damaged replication forks in BS cells [19],[20] and to a limited number of sites of DNA damage in SM-BLM–expressing cells. Thus, multiple factors appear to control RAD51 recruitment to sites of DNA damage. Nonetheless, our findings support the hypothesis that in BLM-expressing cells, BLM SUMOylation promotes RAD51 recruitment and/or retention at sites of DNA damage and thereby facilitates HR-mediated DNA repair.

SUMOylation of BLM is likely to have multiple roles. In addition to regulating its activity at sites of DNA damage as revealed in the current study, BLM SUMOylation may also be important in mediating the localization of BLM to PML-NBs in undamaged cells. SUMO modification can function to retain proteins in the PML-NBs [35], and fluorescence recovery after photobleaching studies have shown that BLM rapidly associates and dissociates from the PML-NBs [52]. This on–off process may be mediated primarily through BLM's SUMO interaction motif, which is required for BLM localization to the PML-NBs and for BLM SUMOylation [53],[54]. The presence of a SUMO binding site(s) in RAD51 suggests that its association with the PML-NBs may also be regulated through the SUMO pathway.

Our results have broad implications for understanding not only how the integrity of replication forks are maintained under stress but also how SUMO modification regulates its substrates, because many proteins in the DNA repair and signaling pathways are SUMO substrates. Although the role of SUMO in HR function is not yet understood, it is clear that SUMOylation plays multiple roles in regulating the HR pathway through modifications of various HR factors, including Sgs1 [32], Rad52 [55]–[57], PCNA [58], and other recombination-associated factors. *sgs1* mutants and mutants of the SUMO-specific E3 ligase gene *mms21* accumulate aberrant cruciform structures at damaged replication forks [32],[33]. This genetic evidence suggests that SUMOylation is important in the regulation of HR, but there has been no direct evidence that SUMOylation occurs at the repair site. Because SUMO-mutant BLM accumulates at HU-induced replication fork damage, the present results indicate that BLM SUMOylation occurs at the sites of damaged replication forks, where it affects stabilization of stalled forks, trafficking of RAD51 to repair sites, and HR repair of damaged forks. Further experiments are now needed to characterize the spatial and temporal regulation of SUMOylation of the different repair factors in HR. In particular, we need to determine what signals activate BLM SUMOylation and how BLM SUMOylation is regulated at damaged forks.

## Materials and Methods

### Antibodies

For BLM Western analysis, rabbit polyclonal anti-BLM antibodies raised against the first 431 amino acids of human BLM [59] or commercially available antibodies (A300-110A, Bethyl Laboratories) were used. Anti-SUMO antibodies were used as described [54]. For indirect immunofluorescence, we used mouse monoclonal anti–γ-H2AX antibody (Upstate), rabbit polyclonal anti-RAD51 antibodies PC130 (Calbiochem), mouse monoclonal anti-PCNA antibody sc-56 (Santa Cruz Biotechnology), Cy-5–labeled donkey anti-rabbit antibodies (Jackson Labs), Alexa Fluor 594–labeled goat anti-mouse, Alexa Fluor 594–labeled goat anti-rabbit, and Alexa Fluor 647–labeled goat anti-mouse antibodies (Invitrogen). Rat monoclonal anti-Hsc70 antibodies (Assay Design) were used as a loading control in Western analyses.

### BLM Expression Constructs and Stable Cell Lines

The full-length BLM cDNA was cloned into the EGFP-C1 vector (Clontech), which produced a GFP-BLM fusion protein with GFP at the N-terminus of BLM, as described previously [53]. The GFP-BLM construct was used as a template for the construction of BLMs that contain SUMO acceptor-site mutations, by substituting arginine for lysine at amino acid residues 317 and 331 using standard polymerase chain reaction–based methods [34]. The construct used in the experiments reported here contained mutations at both 317 and 331. We stably expressed the normal BLM and SUMO-mutant BLM constructs in the SV40-transformed fibroblast cell line GM08505 (BS cells) and isolated multiple clones expressing each construct, as described previously [34],[60]. To measure the levels of GFP-BLM expression, we prepared cell lysates in Laemli sample buffer, fractionated proteins by sodium dodecyl sulphate-polyacrylamide gel electrophoresis, and transferred the proteins to nitrocellulose membranes (Bio-Rad). The membranes were then processed for Western blot analysis and probed with anti-BLM antibodies as described earlier [53]. Varying levels of BLM expression were detected in BLM+ and SM-BLM clones. We chose BLM+ and SM-BLM clones that had comparable levels of transgene expression.

To measure DNA content and percentage of cells in each phase of the cell cycle, cells were harvested by trypsinization and fixed in 70% ethanol for >3 h at −20°C. After fixation, cells were pelleted and resuspended in a solution of 1× phosphate buffered saline (PBS; Gibco) containing propidium iodide (10 µg/ml) and RNase A (0.1 mg/ml). The fluorescence intensities of the propidium iodide–stained cells were measured using a FACScalibur (Becton-Dickinson), and data were analyzed with CellQuest (Becton-Dickinson) and WinMDI (Joe Trotter; http://facs.scripps.edu) software. To examine the percentage of cells in S phase, cells were treated with HU, released, and then analyzed at times after release using the BrdU Flow Kit (BD Biosciences) according to the manufacturer's instructions. BLM+ and SM-BLM cell clones in the logarithmic phase of cell proliferation had comparable proliferation rates, with cell-doubling times equal to ∼30 h for each clone examined.

### Immunofluorescence and Image Analysis

BLM+ and SM-BLM cells were seeded on coverslips and then treated with 10 mM or 0.5 mM HU in culture medium for 1 or 24 h, respectively. To achieve cell synchronization through an independent mechanism, BLM+ and SM-BLM cells were seeded onto coverslips and treated with 0.5 mM mimosine for 24 h. The cells were released into normal medium for 5 h to allow entry into S phase, then treated or not with 10 mM HU for 1 h. For indirect immunofluorescence, cells were washed and fixed at the end of HU treatment. They were then stained with anti-RAD51 and with anti–γ-H2AX or anti-PCNA antibodies, and counterstained with secondary antibodies labeled with Alexa Fluor (Invitrogen). Fixation and staining was performed as described previously [34]. Coverslips were mounted with Prolong Gold antifade reagent containing 4′,6-diamidino-2-phenylindole (DAPI) (Invitrogen). Images were captured on a spinning disk confocal microscope (Carl Zeiss, LSM-510), and data were collected using Slidebook 4.1 software. *Z*-stacks were captured using a 100× oil immersion objective, and the optical slice thickness was 0.2 µm.

A focus was defined as a defined area of the nucleus greater than the minimum area of optical resolution (>0.125 µm^2^) in at least one *Z*-stack in which the fluorescence intensity was greater than the background fluorescence intensity of the nucleoplasm. Colocalization was defined as an area of overlap between two foci of different fluorophores. The maximum number of foci that could be counted in these cells was 150. For purposes of quantification of γ-H2AX foci in the γ-H2AX-bright cells, the γ-H2AX-bright cells were assigned 151 foci, which was one focus more than the maximum countable number. A typical immunofluorescence experiment consisted of assessment of 30–50 cells per condition. The data presented are from two to three independent experiments performed on two or three clones of each type. Immunofluorescence images for figures were created using Image J and Metamorph software (Molecular Devices).

### Flow Cytometry Analysis of γ-H2AX Levels

Cells were untreated or treated with 0.5 mM HU for 24 h. Immediately after treatment, cells were harvested, washed twice with PBS, resuspended in fixation solution (2.3% paraformaldehyde, 0.6% methanol) at a density of approximately 1×10^6^ cells/ml, and incubated on ice for 20 min. Following the fixation process, cells were washed twice with PBS to remove the fixative and were resuspended in permeabilization solution (0.25% saponin, 10 mM HEPES [pH 7.4], 140 mM NaCl, 2.5 mM CaCl_2_) at a concentration of approximately 5×10^6^/ml. Cells were incubated overnight at 4°C in Alexa Fluor 647–conjugated anti-H2A.X (BioLegend). After incubation, the cells were washed twice with 0.1% saponin and 5% fetal bovine serum in PBS and were resuspended in 5% fetal bovine serum in PBS for analysis. Cells were analyzed on a FACScaliber as described above. Data were collected from a minimum of two experiments on five BLM+ clones and six SM-BLM clones. Median fluorescence intensity data was log normalized, and the differences in median intensity were calculated. Log-normalized median intensity differences were tested by Student's *t*-test.

### Measurement of DSBs by PFGE

Cells were untreated or treated with 0.5 mM HU for 24 h and subsequently released into fresh medium for an additional 0, 12, or 24 h, or they were treated with different concentrations of CPT for 3 h. For each damage condition, 4×10^5^ cells were formed into individual 1% agarose plugs (Cleancut agarose, Bio-Rad). The plugs were then incubated in 100 mM EDTA (pH 8.0), 0.2% sodium deoxycholate, 1% sodium lauryl sarcosine, and 1 mg/ml proteinase K at 50°C for 24 h. The plugs were washed four times in 10 mM Tris-HCl (pH 8.0) and 1 mM EDTA for 30 min at room temperature with gentle agitation. Plugs were loaded onto a 0.8% agarose gel (Pulsed Field Certified Agarose, Bio-Rad), and PFGE was preformed on a CHEF DR III (96°, 100°, 106° angle ramp, 1,200–1,800 s switch time, 2 V/cm; Bio-Rad) for 72 h. The gel was stained with SYBR Gold, visualized under UV light, and analyzed using Quantity One and ImageJ software after contrast adjustment. Each lane on the gel was divided into seven areas, and the intensity in each area was analyzed and weighed according to fragment size. The amount of breakage in each lane was further normalized against total DNA content. Values are given relative to the level of DNA breakage in the untreated control. At least two independent experiments were performed on two clones of each type.

### Measurement of HU-Induced Sister-Chromatid Exchanges and Micronuclei Formation

For SCE analyses, cells were cultured with 10 µM BrdU (Sigma-Aldrich). After 60 h, the cells were incubated with 0.02 µg/ml colcemid (Invitrogen) for up to 2 h, harvested and processed as described earlier [60]. The slides were examined under the microscope at 100×, and SCEs were counted from metaphases with an acceptable quality of sister-chromatid discrimination. For measurements of HU-induced SCEs, cells were cultured in 10 µM BrdU for 30 h, washed one time with 1× PBS, and treated with 0.5 mM HU for 24 h. Next, the cells were released into medium containing 10 µM BrdU for an additional 20 h. Metaphases were collected in colcemid and processed as described above. Two independent experiments were performed on two clones of each type.

To quantify the cytogenetic effects of replication-associated DSBs, cells were treated with HU, and micronuclei formation was assessed using the cytokinesis-block micronucleus assay [61],[62]. Cells were plated on chamber slides (Lab-Tek) for approximately 48 h. Next, cells were treated with 0.5 mM HU for 24 h, after which the cells were washed thoroughly with PBS and then incubated in culture medium containing 8.7 µM cytochalasin-B (Sigma-Aldrich). After 28 h of cytochalasin-B treatment, cells were fixed on the slides with a 9∶1 methanol:acetic acid solution and then stained with Diff-Quik (Baxter) according to the manufacturer's instructions. Using a blinded analysis, we examined cells under a light microscope at 100×. A minimum of 500 binucleated cells were assessed under each condition and categorized as follows: cells with no micronucleus, one micronucleus, more than one micronucleus, and nucleoplasmic bridges. Three independent experiments were performed on three clones of each type.

### Cell Death Measurement

To measure cell viability under different DNA damage conditions, 2×10^5^ cells were seeded onto six-well dishes. Cells were treated or not treated with 0.5 mM HU for 24 h and then treated or not treated with 50 µM of etoposide for an additional 24 h. At the end of the second treatment, the cell-growth medium was retained, and the floating cells were combined with adherent cells harvested by trypsinization. We added the ViaCount reagent (Guava Technologies) according to the manufacturer's instructions. Live and dead cells were counted with the Guava Cell Analyzer. A minimum of three independent experiments with three replicates for each condition were performed on three clones of each type.

### Measurement of SUMO-Dependent Binding

Human RAD51 protein was purified as described previously [63]. The purified hRAD51 was either cleaved from the streptavidin-conjugated agarose beads (Ultralink, Pierce) using tobacco etch virus protease or left on the beads and directly used for the binding reactions. SUMO was biotinylated by Sulfo-NHS-Biotin (EZ-link, Pierce) according to the manufacturer's instructions. Equal amounts of unbiotinylated and biotinylated SUMO-1 and SUMO-2 proteins were incubated with RAD51 in binding buffer (PBS with 0.1% Triton X-100) for 2 h at 4°C. Streptavidin beads were then added to each reaction and incubated for another 1 h at 4°C. After five washes with binding buffer, proteins were eluted by SDS-PAGE buffer and analyzed by Western blotting with RAD51 antibodies.

BLM N-terminal fragment (1–431) was modified by SUMO-2 in vitro as described [54]. The SUMO-modified BLM was then aliquoted equally into two tubes with either RAD51-coated streptavidin beads or biotin-coated beads, and rotated in the binding buffer in the presence of 2% BSA at 4°C for 2 h. Unmodified BLM was used as a control. The eluted protein was analyzed by SDS-PAGE and Western blotting with anti-BLM antibodies. Fujifilm Multi Gauge image analysis software (Fujifilm Corp.) was used to determine relative Western blot band intensities and ratios of SUMO-2–modified to unmodified BLM.

### Statistical Analysis

Because observations within each clone may be correlated, we used mixed effects linear models to test the data for statistical significance. In the mixed effects models, each clone was treated as a random effect, and the experimental variables were treated as fixed effects. Because the foci and micronuclei data were not normally distributed, we first applied a square root transformation to stabilize the variance and normalize the data (results in the figures were still presented in the original scale). For testing changes in the number of foci per cell and the number of colocalized foci per cell, cell type (BLM or SM-BLM), treatment (with and without HU), and interaction terms for cell type by treatment were treated as fixed effects. Similarly, for testing changes in the number of micronuclei per cell and the number of SCEs per 46 chromosomes, cell type, treatment with HU, and their interaction were treated as fixed effects. Finally, for testing changes in percentage of cell death, cell type, treatment with HU, treatment with etoposide, and their interaction terms were included as fixed effects. If the random effects (i.e., the clonal variation) were found to be nonsignificant based on likelihood ratio test, the mixed effect models reduced to traditional analysis of variance.

## Supporting Information

Figure S1 **BLM+ and SM-BLM cells have similar cell-cycle profiles.** Representative cell-cycle profiles of cells expressing BLM or SUMO-mutant BLM after HU treatment as determined by BrdU incorporation and flow cytometry analysis. Cells were untreated (NT) or treated with 0.5 mM HU for 24 h. Cells were then analyzed at 0 h, 1 h, 3 h, and 6 h after release from the HU block. Greater than 80% of the cells were in S phase after release from the HU block.
(0.42 MB PDF)

Figure S2 **BS cells accumulate excess DSBs at damaged replication forks.** Quantification of DSBs in untreated BS cells (GM08505) (NT) or cells treated with 0.5 mM HU for 24 h, followed by release into normal medium for 0, 12, and 24 h. Bars represent the numbers of DSBs relative to untreated BS cells in three independent experiments. Error bars represent standard deviation of the data.
(0.01 MB PDF)

Figure S3 **SM-BLM cells are hypersensitive to camptothecin (CPT) compared to BLM+ cells, as determined by colony survival assays.** A total of 1,000–25,000 cells were seeded onto six-well plates overnight, untreated or treated with different concentrations of CPT for 3 h. After treatment, cells were allowed to form colonies in normal medium. Percentage colony survival was calculated as [number of colonies]_treated_/[number of colonies]_untreated_×100. Data represent a single experiment performed on five BLM clones and four SM-BLM clones. GM08505 is the parental BS SV40-transformed human fibroblast cell line and GM00637 is a normal SV40-transformed human fibroblast control; the experiment was performed two times for these cell lines.
(1.88 MB PDF)

Figure S4 **BS cell phenotypes differ from SM-BLM cell phenotypes.** (A) HU induces increased levels of SCEs in BS cells. BS cells (GM08505) were incubated with 10 µM BrdU for 30 h, treated or not with 0.5 mM HU for 24 h, and then returned to BrdU-containing medium for an additional 20 h. Metaphases were collected in colcemid. Two photomicrographs are shown of metaphases in which exchanges between sister chromatids were visualized. Average numbers of SCEs/46 chromosomes for untreated and HU-treated BS cells is shown beneath the photomicrographs (number of metaphases counted). (B) HU-treated BS cells (GM08505) exhibit excess colocalization of RAD51 and γ-H2AX, demonstrating that RAD51 is effectively recruited to damaged replication forks in the absence of BLM. Bars indicate 10 µm.
(5.80 MB PDF)

Figure S5 **Graphical representation of mean numbers of colocalized RAD51 and BLM foci in untreated (NT) and HU-treated (HU) BLM+ and SM-BLM cells.**
(0.01 MB PDF)

Figure S6 **Impaired localization of RAD51 to damaged forks in SUMO-mutant BLM cells after a short treatment with HU.** BLM+ and SM-BLM cells were untreated (NT) or synchronized with 0.5 mM mimosine for 24 h, which stalls cells in late G1 phase, released into normal medium for 5 h to allow the cells to enter S phase, then treated (MIM+HU) or not (MIM rel) with 10 mM HU for 1 h and evaluated for RAD51 localization with BLM and γ-H2AX. BrdU flow cytometry, as performed in Figure S1, confirmed that >80% of the cells had entered S phase by 5 h after release. Data presented are the average of two experiments in each of two BLM+ and two SM-BLM clones. Bars represent the mean numbers of foci or colocalized foci per nucleus, and the error bars represent the standard deviations of the combined data. After 1 h of treatment with 10 mM HU, RAD51 foci increased in BLM+ cells but were effectively unchanged in SM-BLM cells. These effects on RAD51 localization are observed despite the fact that mimosine treatment induces many γ-H2AX foci, which has been previously reported [64].
(0.01 MB PDF)

Figure S7 **BLM and SM-BLM localize to stalled replication forks with similar efficiency.** (A) Immunofluorescence images of representative S-phase BLM+ and SM-BLM cells treated with 0.5 mM HU for 24 h. Cells were stained with antibodies to PCNA. Images show GFP-BLM fluorescence, PCNA staining, and merged BLM-PCNA immunofluorescence. (B) Graphical representation of the average numbers of BLM foci, PCNA foci, and colocalized BLM-PCNA foci in HU-treated BLM+ and SM-BLM cells. The numbers of BLM and PCNA foci were counted in cells that stained positively for PCNA, as these represented cells in S phase. Data presented are the average of two experiments in each of two BLM+ and two SM-BLM clones. Bars represent the mean numbers of foci or colocalized foci per nucleus, and the error bars represent the standard deviations of the combined data. The levels of BLM foci and PCNA foci were higher in SM-BLM than in BLM+ cells; however, the percentages of colocalized BLM-PCNA foci were similar in BLM+ and SM-BLM cells, indicating that SUMO-mutant BLM localizes normally to sites of replication damage.
(0.99 MB PDF)

## Abbreviations

BS: Bloom's syndrome
DSB: double-strand break
HR: homologous recombination
PFGE: pulsed-field gel electrophoresis
PML-NB: promyelocytic leukemia nuclear body
SCE: sister-chromatid exchange
ssDNA: single-stranded DNA

## References

[1] Kowalczykowski S. C (2000). Initiation of genetic recombination and recombination-dependent replication.. Trends Biochem Sci.

[2] Wilson D. M, Thompson L. H (2007). Molecular mechanisms of sister-chromatid exchange.. Mutat Res.

[3] Heartlein M. W, Tsuji H, Latt S. A (1987). 5-Bromodeoxyuridine-dependent increase in sister chromatid exchange formation in Bloom's syndrome is associated with reduction in topoisomerase II activity.. Exp Cell Res.

[4] Palitti F, Tanzarella C, Degrassi F, De Salvia R, Fiore M (1984). Enhancement of induced sister chromatid exchange and chromosomal aberrations by inhibitors of DNA repair processes.. Toxicol Pathol.

[5] Popescu N. C, Turnbull D, DiPaolo J. A (1977). Sister chromatid exchange and chromosome aberration analysis with the use of several carcinogens and noncarcinogens.. J Natl Cancer Inst.

[6] Chaganti R. S, Schonberg S, German J (1974). A manyfold increase in sister chromatid exchanges in Bloom's syndrome lymphocytes.. Proc Natl Acad Sci U S A.

[7] Woo L. L, Onel K, Ellis N. A (2007). The broken genome: genetic and pharmacologic approaches to breaking DNA.. Ann Med.

[8] German J, Sanz M. M, Ciocci S, Ye T. Z, Ellis N. A (2007). Syndrome-causing mutations of the BLM gene in persons in the Bloom's Syndrome Registry.. Hum Mutat.

[9] Ouyang K. J, Woo L. L, Ellis N. A (2008). Homologous recombination and maintenance of genome integrity: Cancer and aging through the prism of human RecQ helicases.. Mech Ageing Dev.

[10] Chu W. K, Hickson I. D (2009). RecQ helicases: multifunctional genome caretakers.. Nat Rev Cancer.

[11] Raynard S, Bussen W, Sung P (2006). A double Holliday junction dissolvasome comprising BLM, topoisomerase IIIalpha, and BLAP75.. J Biol Chem.

[12] Wu L, Hickson I. D (2003). The Bloom's syndrome helicase suppresses crossing over during homologous recombination.. Nature.

[13] Wu L, Bachrati C. Z, Ou J, Xu C, Yin J (2006). BLAP75/RMI1 promotes the BLM-dependent dissolution of homologous recombination intermediates.. Proc Natl Acad Sci U S A.

[14] Davalos A. R, Campisi J (2003). Bloom syndrome cells undergo p53-dependent apoptosis and delayed assembly of BRCA1 and NBS1 repair complexes at stalled replication forks.. J Cell Biol.

[15] Li W, Kim S. M, Lee J, Dunphy W. G (2004). Absence of BLM leads to accumulation of chromosomal DNA breaks during both unperturbed and disrupted S phases.. J Cell Biol.

[16] Sengupta S, Robles A. I, Linke S. P, Sinogeeva N. I, Zhang R (2004). Functional interaction between BLM helicase and 53BP1 in a Chk1-mediated pathway during S-phase arrest.. J Cell Biol.

[17] Bjergbaek L, Cobb J. A, Tsai-Pflugfelder M, Gasser S. M (2005). Mechanistically distinct roles for Sgs1p in checkpoint activation and replication fork maintenance.. EMBO J.

[18] Cobb J. A, Bjergbaek L, Shimada K, Frei C, Gasser S. M (2003). DNA polymerase stabilization at stalled replication forks requires Mec1 and the RecQ helicase Sgs1.. EMBO J.

[19] Bischof O, Kim S. H, Irving J, Beresten S, Ellis N. A (2001). Regulation and localization of the Bloom syndrome protein in response to DNA damage.. J Cell Biol.

[20] Wu L, Davies S. L, Levitt N. C, Hickson I. D (2001). Potential role for the BLM helicase in recombinational repair via a conserved interaction with RAD51.. J Biol Chem.

[21] Bugreev D. V, Yu X, Egelman E. H, Mazin A. V (2007). Novel pro- and anti-recombination activities of the Bloom's syndrome helicase.. Genes Dev.

[22] van Brabant A. J, Ye T, Sanz M, German J. L, Ellis N. A (2000). Binding and melting of D-loops by the Bloom syndrome helicase.. Biochemistry.

[23] Gravel S, Chapman J. R, Magill C, Jackson S. P (2008). DNA helicases Sgs1 and BLM promote DNA double-strand break resection.. Genes Dev.

[24] Mimitou E. P, Symington L. S (2008). Sae2, Exo1 and Sgs1 collaborate in DNA double-strand break processing.. Nature.

[25] Nimonkar A. V, Ozsoy A. Z, Genschel J, Modrich P, Kowalczykowski S. C (2008). Human exonuclease 1 and BLM helicase interact to resect DNA and initiate DNA repair.. Proc Natl Acad Sci U S A.

[26] Zhu Z, Chung W. H, Shim E. Y, Lee S. E, Ira G (2008). Sgs1 helicase and two nucleases Dna2 and Exo1 resect DNA double-strand break ends.. Cell.

[27] Johnson E. S (2004). Protein modification by SUMO.. Annu Rev Biochem.

[28] Papouli E, Chen S, Davies A. A, Huttner D, Krejci L (2005). Crosstalk between SUMO and ubiquitin on PCNA is mediated by recruitment of the helicase Srs2p.. Mol Cell.

[29] Hoege C, Pfander B, Moldovan G. L, Pyrowolakis G, Jentsch S (2002). RAD6-dependent DNA repair is linked to modification of PCNA by ubiquitin and SUMO.. Nature.

[30] Pfander B, Moldovan G. L, Sacher M, Hoege C, Jentsch S (2005). SUMO-modified PCNA recruits Srs2 to prevent recombination during S phase.. Nature.

[31] Stelter P, Ulrich H. D (2003). Control of spontaneous and damage-induced mutagenesis by SUMO and ubiquitin conjugation.. Nature.

[32] Branzei D, Sollier J, Liberi G, Zhao X, Maeda D (2006). Ubc9- and mms21-mediated sumoylation counteracts recombinogenic events at damaged replication forks.. Cell.

[33] Liberi G, Maffioletti G, Lucca C, Chiolo I, Baryshnikova A (2005). Rad51-dependent DNA structures accumulate at damaged replication forks in sgs1 mutants defective in the yeast ortholog of BLM RecQ helicase.. Genes Dev.

[34] Eladad S, Ye T. Z, Hu P, Leversha M, Beresten S (2005). Intra-nuclear trafficking of the BLM helicase to DNA damage-induced foci is regulated by SUMO modification.. Hum Mol Genet.

[35] Matunis M. J, Zhang X. D, Ellis N. A (2006). SUMO: the glue that binds.. Dev Cell.

[36] Avemann K, Knippers R, Koller T, Sogo J. M (1988). Camptothecin, a specific inhibitor of type I DNA topoisomerase, induces DNA breakage at replication forks.. Mol Cell Biol.

[37] Hsiang Y. H, Lihou M. G, Liu L. F (1989). Arrest of replication forks by drug-stabilized topoisomerase I-DNA cleavable complexes as a mechanism of cell killing by camptothecin.. Cancer Res.

[38] Strumberg D, Pilon A. A, Smith M, Hickey R, Malkas L (2000). Conversion of topoisomerase I cleavage complexes on the leading strand of ribosomal DNA into 5′-phosphorylated DNA double-strand breaks by replication runoff.. Mol Cell Biol.

[39] Chan K. L, North P. S, Hickson I. D (2007). BLM is required for faithful chromosome segregation and its localization defines a class of ultrafine anaphase bridges.. EMBO J.

[40] Chan K. L, Palmai-Pallag T, Ying S, Hickson I. D (2009). Replication stress induces sister-chromatid bridging at fragile site loci in mitosis.. Nat Cell Biol.

[41] Davalos A. R, Kaminker P, Hansen R. K, Campisi J (2004). ATR and ATM-dependent movement of BLM helicase during replication stress ensures optimal ATM activation and 53BP1 focus formation.. Cell Cycle.

[42] Davies S. L, North P. S, Dart A, Lakin N. D, Hickson I. D (2004). Phosphorylation of the Bloom's syndrome helicase and its role in recovery from S-phase arrest.. Mol Cell Biol.

[43] Rao V. A, Fan A. M, Meng L, Doe C. F, North P. S (2005). Phosphorylation of BLM, dissociation from topoisomerase IIIalpha, and colocalization with gamma-H2AX after topoisomerase I-induced replication damage.. Mol Cell Biol.

[44] Davies S. L, North P. S, Hickson I. D (2007). Role for BLM in replication-fork restart and suppression of origin firing after replicative stress.. Nat Struct Mol Biol.

[45] Rao V. A, Conti C, Guirouilh-Barbat J, Nakamura A, Miao Z. H (2007). Endogenous gamma-H2AX-ATM-Chk2 checkpoint activation in Bloom's syndrome helicase deficient cells is related to DNA replication arrested forks.. Mol Cancer Res.

[46] Groden J, Nakamura Y, German J (1990). Molecular evidence that homologous recombination occurs in proliferating human somatic cells.. Proc Natl Acad Sci U S A.

[47] Adams M. D, McVey M, Sekelsky J. J (2003). Drosophila BLM in double-strand break repair by synthesis-dependent strand annealing.. Science.

[48] Magner D. B, Blankschien M. D, Lee J. A, Pennington J. M, Lupski J. R (2007). RecQ promotes toxic recombination in cells lacking recombination intermediate-removal proteins.. Mol Cell.

[49] Lallemand-Breitenbach V, Jeanne M, Benhenda S, Nasr R, Lei M (2008). Arsenic degrades PML or PML-RARalpha through a SUMO-triggered RNF4/ubiquitin-mediated pathway.. Nat Cell Biol.

[50] Sun H, Leverson J. D, Hunter T (2007). Conserved function of RNF4 family proteins in eukaryotes: targeting a ubiquitin ligase to SUMOylated proteins.. EMBO J.

[51] Tatham M. H, Geoffroy M. C, Shen L, Plechanovova A, Hattersley N (2008). RNF4 is a poly-SUMO-specific E3 ubiquitin ligase required for arsenic-induced PML degradation.. Nat Cell Biol.

[52] Weidtkamp-Peters S, Lenser T, Negorev D, Gerstner N, Hofmann T. G (2008). Dynamics of component exchange at PML nuclear bodies.. J Cell Sci.

[53] Hu P, Beresten S. F, van Brabant A. J, Ye T. Z, Pandolfi P. P (2001). Evidence for BLM and Topoisomerase IIIalpha interaction in genomic stability.. Hum Mol Genet.

[54] Zhu J, Zhu S, Guzzo C. M, Ellis N. A, Sung K. S (2008). Small ubiquitin-related modifier (SUMO) binding determines substrate recognition and paralog-selective SUMO modification.. J Biol Chem.

[55] Burgess R. C, Rahman S, Lisby M, Rothstein R, Zhao X (2007). The Slx5-Slx8 complex affects sumoylation of DNA repair proteins and negatively regulates recombination.. Mol Cell Biol.

[56] Ohuchi T, Seki M, Branzei D, Maeda D, Ui A (2008). Rad52 sumoylation and its involvement in the efficient induction of homologous recombination.. DNA Repair.

[57] Sacher M, Pfander B, Hoege C, Jentsch S (2006). Control of Rad52 recombination activity by double-strand break-induced SUMO modification.. Nat Cell Biol.

[58] Moldovan G. L, Pfander B, Jentsch S (2007). PCNA, the maestro of the replication fork.. Cell.

[59] Beresten S. F, Stan R, van Brabant A. J, Ye T, Naureckiene S (1999). Purification of overexpressed hexahistidine-tagged BLM N431 as oligomeric complexes.. Protein Expr Purif.

[60] Ellis N. A, Proytcheva M, Sanz M. M, Ye T. Z, German J (1999). Transfection of BLM into cultured bloom syndrome cells reduces the sister-chromatid exchange rate toward normal.. Am J Hum Genet.

[61] Fenech M (2006). Cytokinesis-block micronucleus assay evolves into a “cytome” assay of chromosomal instability, mitotic dysfunction and cell death.. Mutat Res.

[62] Fenech M (2007). Cytokinesis-block micronucleus cytome assay.. Nat Protoc.

[63] Jayathilaka K, Sheridan S. D, Bold T. D, Bochenska K, Logan H. L (2008). A chemical compound that stimulates the human homologous recombination protein RAD51.. Proc Natl Acad Sci U S A.

[64] Saintigny Y, Delacôte F, Varès G, Petitot F, Lambert S (2001). Characterization of homologous recombination induced by replication inhibition in mammalian cells.. EMBO J.

