# Supplementary figures and images for: SUMO Modification Regulates BLM and RAD51 Interaction at Damaged Replication Forks
Source: PLoS Biol. 2009 Dec 1;7(12):e1000252. doi: 10.1371/journal.pbio.1000252 (PMC2779653; doi:10.1371/journal.pbio.1000252)

**Figure S1**

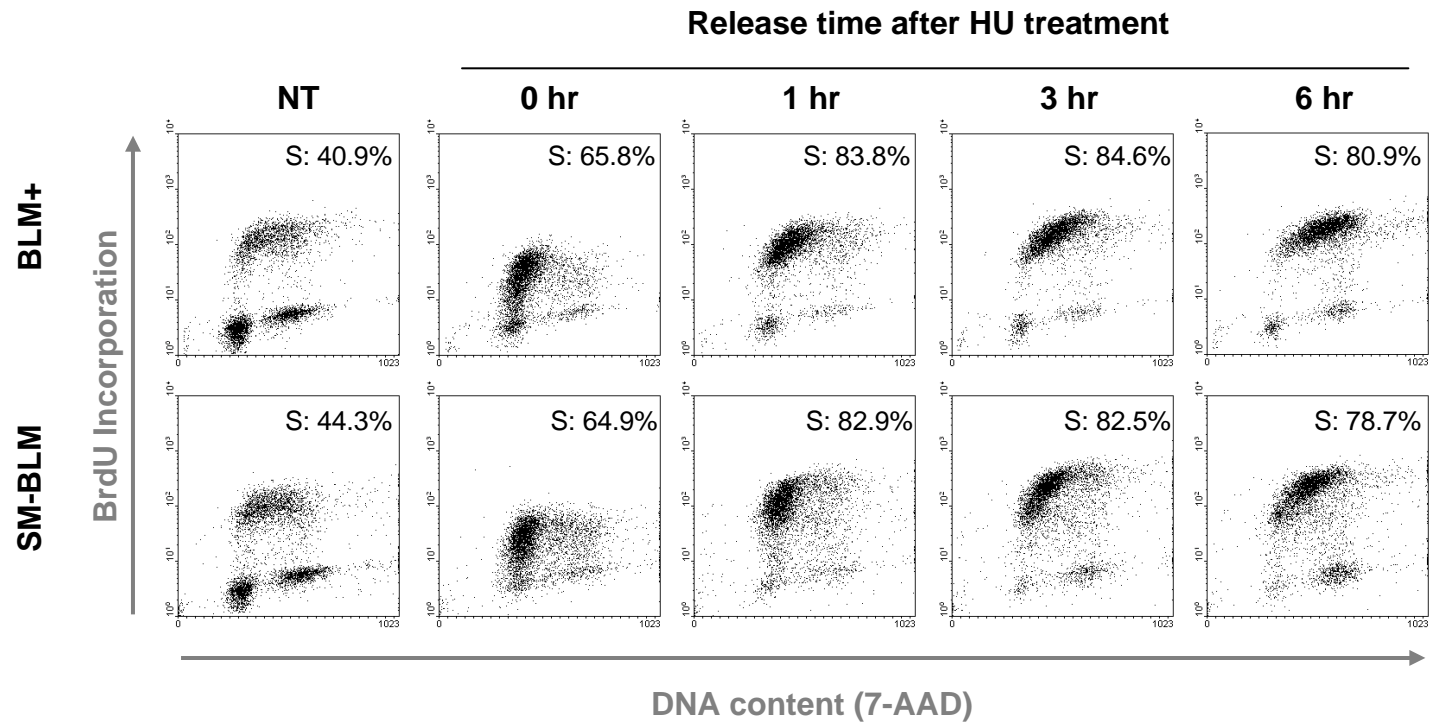

Supplement: Figure S1 — BLM+ and SM-BLM cells have similar cell-cycle profiles. Representative cell-cycle profiles of cells expressing BLM or SUMO-mutant BLM after HU treatment as determined by BrdU incorporation and flow cytometry analysis. Cells were untreated (NT) or treated with 0.5 mM HU for 24 h. Cells were then analyzed at 0 h, 1 h, 3 h, and 6 h after release from the HU block. Greater than 80% of the cells were in S phase after release from the HU block. (0.42 MB PDF) [file pbio.1000252.s001.pdf]

**Figure S2**

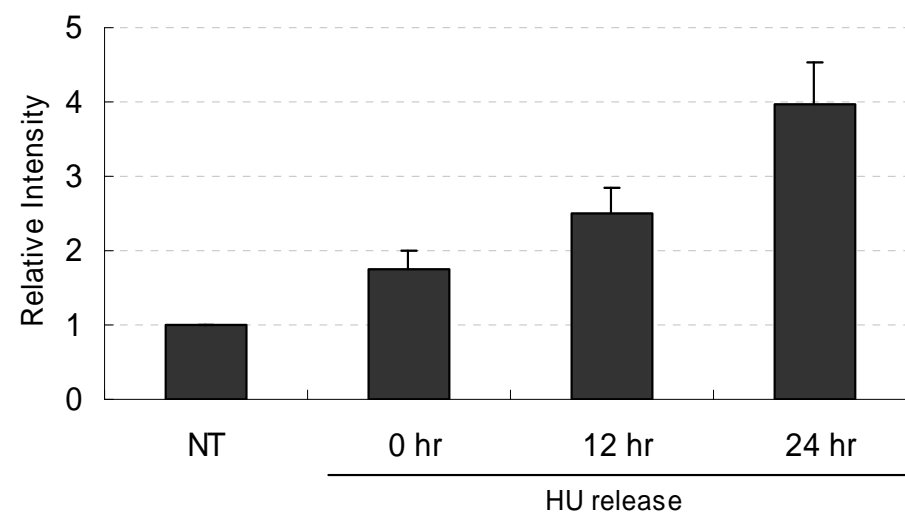

Supplement: Figure S2 — BS cells accumulate excess DSBs at damaged replication forks. Quantification of DSBs in untreated BS cells (GM08505) (NT) or cells treated with 0.5 mM HU for 24 h, followed by release into normal medium for 0, 12, and 24 h. Bars represent the numbers of DSBs relative to untreated BS cells in three independent experiments. Error bars represent standard deviation of the data. (0.01 MB PDF) [file pbio.1000252.s002.pdf]

**Figure S3**

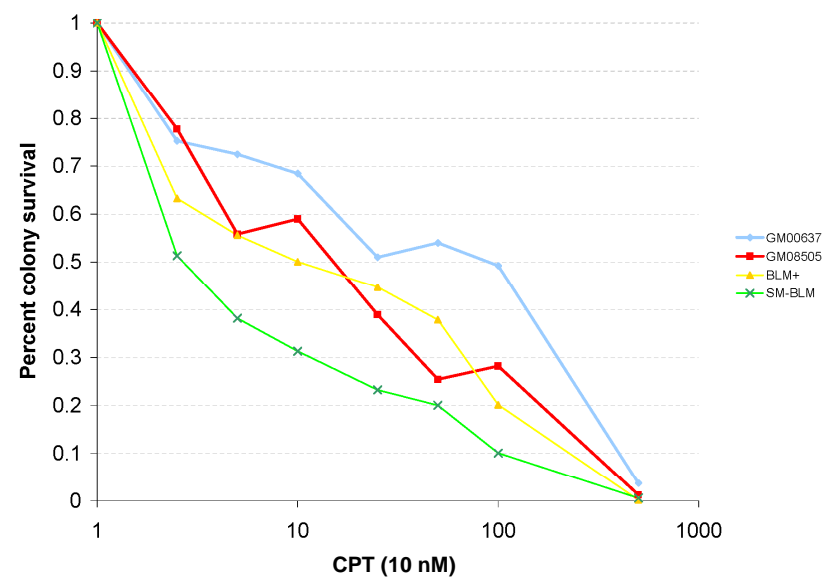

Supplement: Figure S3 — SM-BLM cells are hypersensitive to camptothecin (CPT) compared to BLM+ cells, as determined by colony survival assays. A total of 1,000–25,000 cells were seeded onto six-well plates overnight, untreated or treated with different concentrations of CPT for 3 h. After treatment, cells were allowed to form colonies in normal medium. Percentage colony survival was calculated as [number of colonies]treated/[number of colonies]untreated×100. Data represent a single experiment performed on five BLM clones and four SM-BLM clones. GM08505 is the parental BS SV40-transformed human fibroblast cell line and GM00637 is a normal SV40-transformed human fibroblast control; the experiment was performed two times for these cell lines. (1.88 MB PDF) [file pbio.1000252.s003.pdf]

**Figure S4**

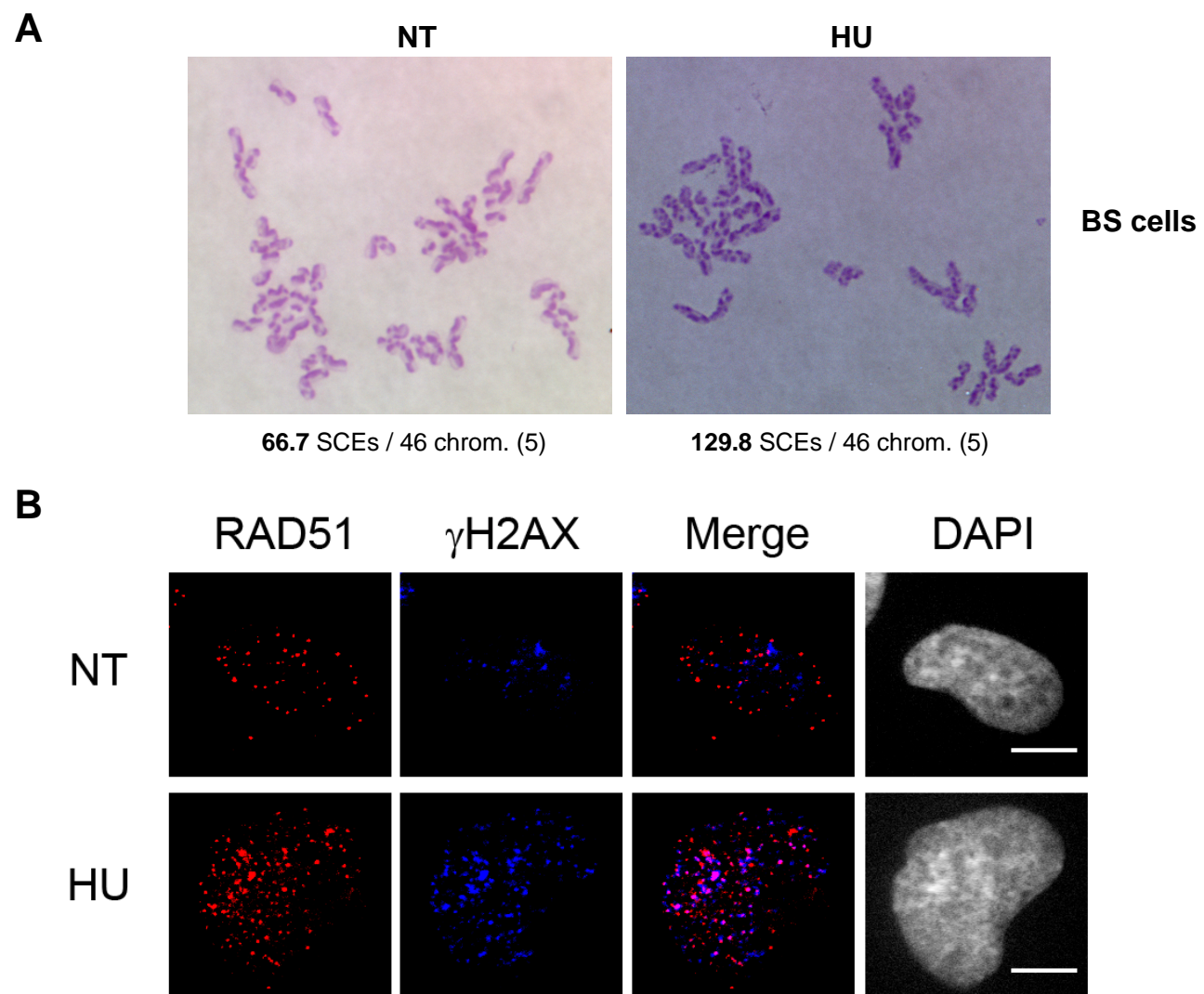

Supplement: Figure S4 — BS cell phenotypes differ from SM-BLM cell phenotypes. (A) HU induces increased levels of SCEs in BS cells. BS cells (GM08505) were incubated with 10 µM BrdU for 30 h, treated or not with 0.5 mM HU for 24 h, and then returned to BrdU-containing medium for an additional 20 h. Metaphases were collected in colcemid. Two photomicrographs are shown of metaphases in which exchanges between sister chromatids were visualized. Average numbers of SCEs/46 chromosomes for untreated and HU-treated BS cells is shown beneath the photomicrographs (number of metaphases counted). (B) HU-treated BS cells (GM08505) exhibit excess colocalization of RAD51 and γ-H2AX, demonstrating that RAD51 is effectively recruited to damaged replication forks in the absence of BLM. Bars indicate 10 µm. (5.80 MB PDF) [file pbio.1000252.s004.pdf]

**Figure S5**

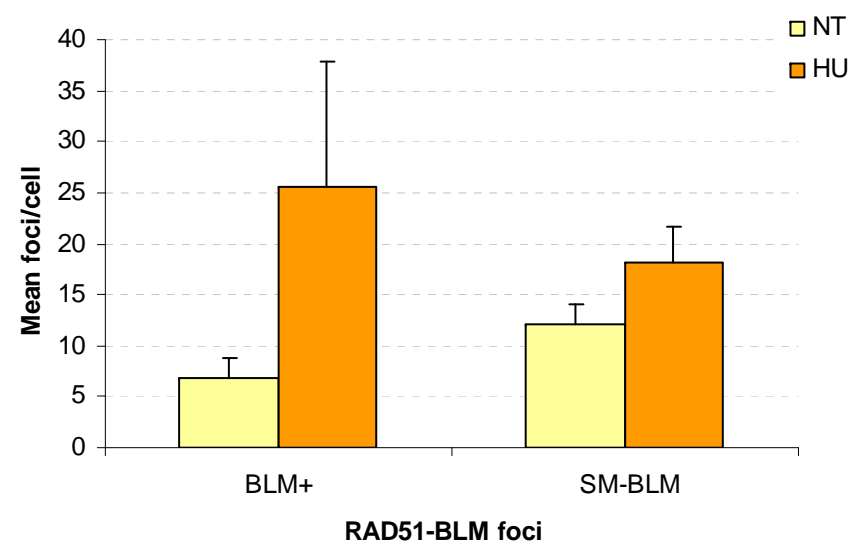

Supplement: Figure S5 — Graphical representation of mean numbers of colocalized RAD51 and BLM foci in untreated (NT) and HU-treated (HU) BLM+ and SM-BLM cells. (0.01 MB PDF) [file pbio.1000252.s005.pdf]

**Figure S6**

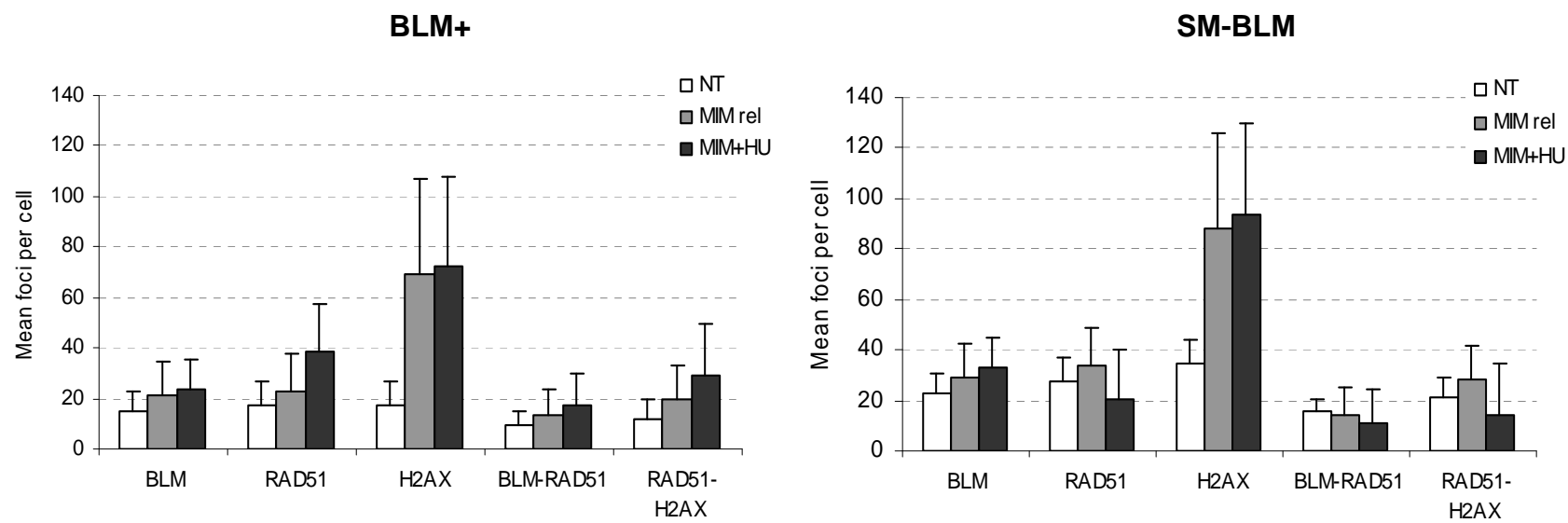

Supplement: Figure S6 — Impaired localization of RAD51 to damaged forks in SUMO-mutant BLM cells after a short treatment with HU. BLM+ and SM-BLM cells were untreated (NT) or synchronized with 0.5 mM mimosine for 24 h, which stalls cells in late G1 phase, released into normal medium for 5 h to allow the cells to enter S phase, then treated (MIM+HU) or not (MIM rel) with 10 mM HU for 1 h and evaluated for RAD51 localization with BLM and γ-H2AX. BrdU flow cytometry, as performed in Figure S1, confirmed that >80% of the cells had entered S phase by 5 h after release. Data presented are the average of two experiments in each of two BLM+ and two SM-BLM clones. Bars represent the mean numbers of foci or colocalized foci per nucleus, and the error bars represent the standard deviations of the combined data. After 1 h of treatment with 10 mM HU, RAD51 foci increased in BLM+ cells but were effectively unchanged in SM-BLM cells. These effects on RAD51 localization are observed despite the fact that mimosine treatment induces many γ-H2AX foci, which has been previously reported [64]. (0.01 MB PDF) [file pbio.1000252.s006.pdf]

**Figure S7**

**A**

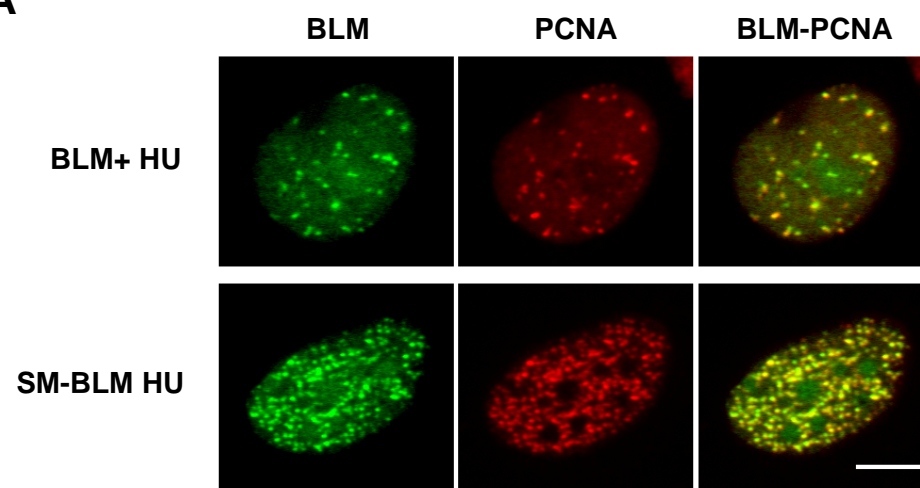

**B**

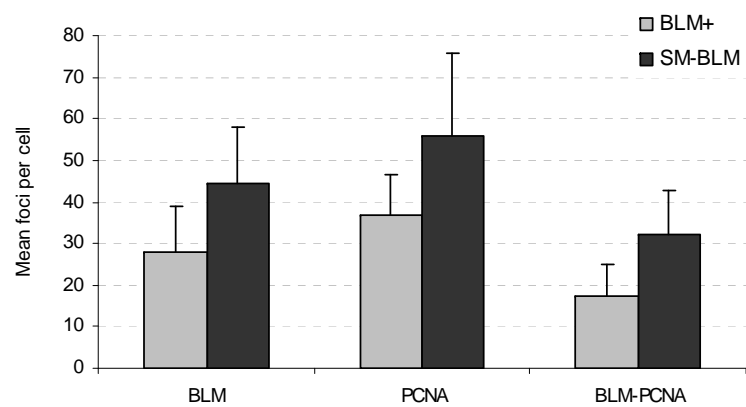

Supplement: Figure S7 — BLM and SM-BLM localize to stalled replication forks with similar efficiency. (A) Immunofluorescence images of representative S-phase BLM+ and SM-BLM cells treated with 0.5 mM HU for 24 h. Cells were stained with antibodies to PCNA. Images show GFP-BLM fluorescence, PCNA staining, and merged BLM-PCNA immunofluorescence. (B) Graphical representation of the average numbers of BLM foci, PCNA foci, and colocalized BLM-PCNA foci in HU-treated BLM+ and SM-BLM cells. The numbers of BLM and PCNA foci were counted in cells that stained positively for PCNA, as these represented cells in S phase. Data presented are the average of two experiments in each of two BLM+ and two SM-BLM clones. Bars represent the mean numbers of foci or colocalized foci per nucleus, and the error bars represent the standard deviations of the combined data. The levels of BLM foci and PCNA foci were higher in SM-BLM than in BLM+ cells; however, the percentages of colocalized BLM-PCNA foci were similar in BLM+ and SM-BLM cells, indicating that SUMO-mutant BLM localizes normally to sites of replication damage. (0.99 MB PDF) [file pbio.1000252.s007.pdf]
